# ^18^F-FDG PET/CT Radiomic Analysis with Machine Learning for Identifying Bone Marrow Involvement in the Patients with Suspected Relapsed Acute Leukemia

**DOI:** 10.7150/thno.33841

**Published:** 2019-07-09

**Authors:** Hebei Li, Chongrui Xu, Bowen Xin, Chaojie Zheng, Yunyun Zhao, Keji Hao, Qian Wang, Richard L. Wahl, Xiuying Wang, Yun Zhou

**Affiliations:** 1 Department of Nuclear Medicine, Peking University People's Hospital, Beijing 100044, China; 2 Mallinckrodt Institute of Radiology, Washington University in St. Louis School of Medicine, St. Louis, MO 63110, United States of America; 3 School of Computer Science, the University of Sydney, NSW 2006, Australia

**Keywords:** ^ 18^F-FDG PET/CT, relapsed leukemia, bone marrow involvement, radiomics, machine learning

## Abstract

^18^F-FDG PET / CT is used clinically for the detection of extramedullary lesions in patients with relapsed acute leukemia (AL). However, the visual analysis of ^18^F-FDG diffuse bone marrow uptake in detecting bone marrow involvement (BMI) in routine clinical practice is still challenging. This study aims to improve the diagnostic performance of ^18^F-FDG PET/CT in detecting BMI for patients with suspected relapsed AL.

**Methods**: Forty-one patients (35 in training group and 6 in independent validation group) with suspected relapsed AL were retrospectively included in this study. All patients underwent both bone marrow biopsy (BMB) and ^18^F-FDG PET/CT within one week. The BMB results were used as the gold standard or real “truth” for BMI. The bone marrow ^18^F-FDG uptake was visually diagnosed as positive and negative by three nuclear medicine physicians. The skeletal volumes of interest were manually drawn on PET/CT images. A total of 781 PET and 1045 CT radiomic features were automatically extracted to provide a more comprehensive understanding of the embedded pattern. To select the most important and predictive features, an unsupervised consensus clustering method was first performed to analyze the feature correlations and then used to guide a random forest supervised machine learning model for feature importance analysis. Cross-validation and independent validation were conducted to justify the performance of our model.

**Results**: The training group involved 16 BMB positive and 19 BMB negative patients. Based on the visual analysis of ^18^F-FDG PET, 3 patients had focal uptake, 8 patients had normal uptake, and 24 patients had diffuse uptake. The sensitivity, specificity, and accuracy of visual analysis for BMI diagnosis were 62.5%, 73.7%, and 68.6%, respectively. With the cross-validation on the training group, the machine learning model correctly predicted 31 patients in BMI. The sensitivity, specificity, and accuracy of the machine learning model in BMI detection were 87.5%, 89.5%, and 88.6%, respectively, significantly higher than the ones in visual analysis (*P* < 0.05). The evaluation on the independent validation group showed that the machine learning model could achieve 83.3% accuracy.

**Conclusions:** ^18^F-FDG PET/CT radiomic analysis with machine learning model provided a quantitative, objective and efficient mechanism for identifying BMI in the patients with suspected relapsed AL. It is suggested in particular for the diagnosis of BMI in the patients with ^18^F-FDG diffuse uptake patterns.

## Introduction

Acute leukemia (AL) is a hematological malignancy characterized by a rapid increase in the number of immature blood cells. Despite the high rates of initial complete remission, relapse remains a formidable clinical challenge and has become a major cause of failure in treatment 1. Leukemia relapse can occur intramedullary or extramedullary, or both. Patients typically undergo multiple bone marrow biopsy (BMB) in the follow-up to monitor the intramedullary relapse 2. However, BMB is an invasive test and only evaluates a small proportion of the entire bone marrow. ^18^F-fluorodesoxyglucose positron emission tomography/computed tomography (^18^F-FDG PET/CT) has been proven to detect more extramedullary lesions missed by routine examinations 3-8.

The diagnosis of ^18^F-FDG PET/CT-based leukemic bone marrow involvement (BMI) has not been fully evaluated due to the lack of systematic and large-scale studies. From the available leukemic bone marrow studies, mostly are case reports, we could speculate that diffuse uptake is the major pattern 9-12, and its incidence is much higher than that in the lymphoma studies 13. It is quite difficult to determine whether diffuse uptake is BMI in visual assessment, because the judgment depends on the physician's experience, and both malignant and benign causes may have similar appearance 11, 14, 15. In some lymphomatous bone marrow studies, diffuse uptake was considered to be BMI negative 16, 17, while in other studies it was considered as BMI positive 18, 19. Because of the relatively high incidence of diffuse uptake in leukemia patients, it is not appropriate to take diffuse uptake as positive or negative for BMI in patients with suspected relapsed AL. In summary, the clinical ^18^F-FDG PET/CT-based diagnosis of BMI in relapsed AL is still challenging.

Radiomics extracted and mined a large number of medical imaging features to quantify tumor phenotypic characteristics and could reveal features of the disease that are incomprehensible to the naked eye. It has been used in many solid tumors 20-22, while rarely used in bone marrow assessment. A recently published study indicated that ^18^F-FDG PET-based radiomic analysis was helpful in identifying BMI 23. We hypothesize that high-dimensional, high-throughput radiomic features from both PET and CT images would provide a thorough strategy for extracting the pattern of BMI, and thereby would be helpful in improving the diagnostic power of ^18^F-FDG PET/CT in patients with suspected relapsed AL.

## Materials and Methods

### Patients

The study has been approved by the institution review board, and the need for written informed consent was waived. This study retrospectively analyzed images of AL patients who underwent ^18^F-FDG PET/CT at Peking University People's Hospital between January 2012 and February 2019. The inclusion criteria were as follows: 1) acute myeloid leukemia or acute lymphoblastic leukemia patients who achieved complete remission after induction chemotherapy, 2) Age ≥ 16, 3) clinically suspected recurrence, but not yet started treatment, 4) no chemotherapy or granulocyte stimulation-factor within 1 month, 5) BMB has been completed within 1 week. The simple statistics of selected patients are summarized in **Table 1**. The patients were divided into two groups, i.e. 35 patients from January 2012 to February 2018 as training group and 6 patients from March 2018 to February 2019 as independent validation group.

### PET/CT acquisition and reconstruction parameters

All patients fasted at least 6 h before scan, and the blood glucose level were controlled below 8.3 mM (range 4.7~8.0 mM). ^18^F-FDG (provided by Atom high-tech Co., Ltd., Beijing, China) was injected intravenously with a weight-base dose of 5.55 MBq/kg (0.15 mCi/kg). After 60 minutes (60 ± 5 min, range 54 ~63 min) ^18^F-FDG injection, the PET scan between the base of skull and the middle of the thigh was performed on a Discovery VCT (GE Healthcare, Milwaukee, Wisconsin, USA) with a 64-slice spiral CT. CT scan was firstly performed with a tube voltage of 140 Kev and a tube current of 80 mAs. The matrix size of CT was 512 × 512 with the voxel size 1.0 × 1.0 × 3.3 mm^3^. The PET data were collected in 3D mode for 2.5 min/bed and were corrected for attenuation with a CT-based attenuation correction method. The PET images were reconstructed using an iterative algorithm (ordered-subset expectation maximization with 2 iterations, 28 subsets) and 6-mm full width at half maximum (FWHM) of Gaussian filter. The matrix size of PET was 128 × 128 with the voxel size 5.5 × 5.5 × 3.3 mm^3^.

### Clinical PET/CT review

Three nuclear medicine physicians with 15, 10, and 10 years of PET/CT reading experiences visually assessed bone marrow ^18^F-FDG uptake in each patient. They were allowed to refer the corresponding clinical data except for the BMB results. Focal uptake, the presence of ^18^F-FDG-avid foci, which could not be explained by benign findings on underlying CT or clinical history, was considered as positive for BMI. Normal uptake, the uptake of bone marrow equal to or lower than the liver, was considered as negative for BMI. For the diffuse uptake, the uptake of bone marrow higher than liver, the physicians made their diagnosis based on their visual assessment in the ^18^F-FDG bone marrow uptake distribution, intensity and apparent heterogeneity.

In case of discrepancy, the examination was conjointly reviewed to reach a consensus. The BMB results were used as the gold standard or real “truth” for BMI diagnosis in the study. All the true positives (TP) and true negatives (TN) were recorded as successful diagnosis, whereas all the false positives (FP) and false negatives (FN) cases were recorded as failed diagnosis.

### PET/CT radiomic analysis with machine learning

**Figure 1:**
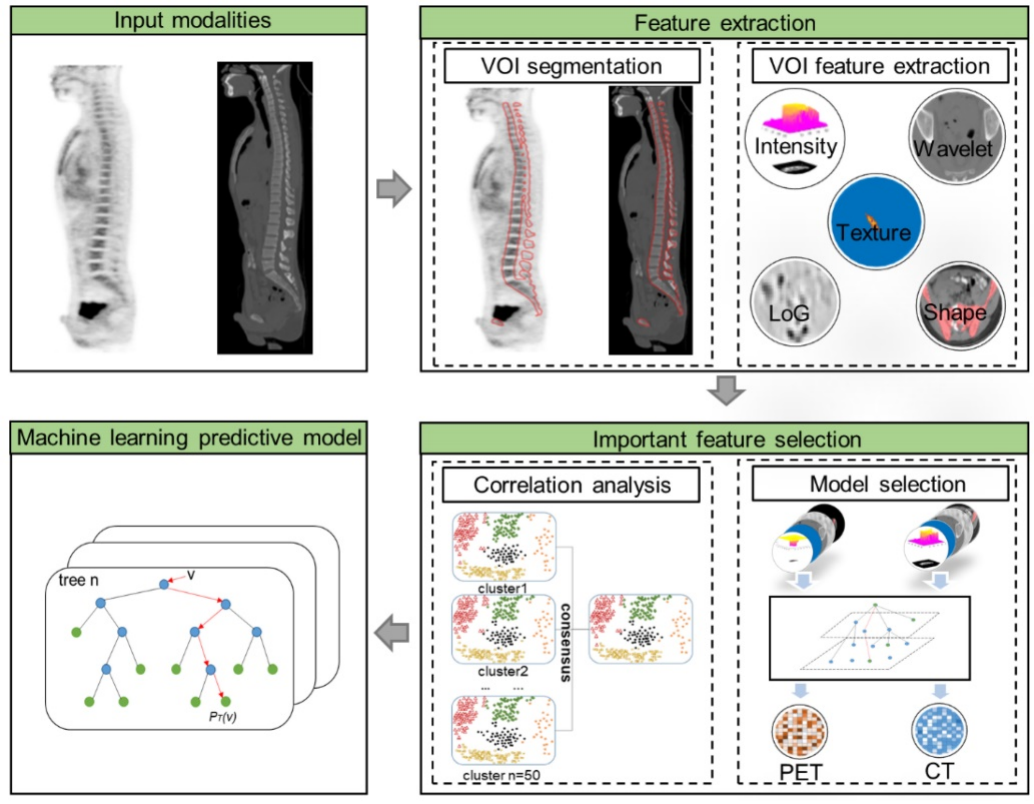
The flow chart of radiomic features extraction and selection.

As illustrated in **Figure 1**, the radiomic analysis composed of three major stages. Firstly, based on the manual delineation of the volumes of interest (VOIs) from CT and then ascertained on PET, our model automatically extracted high-dimensional imaging features from both PET and CT VOIs; then important and discriminative features for pattern extraction were selected using harnessed correlation analysis and machine learning models; and finally, a machine learning based prediction model was validated for the classification of BMB cases.

The first stage was radiomic feature extraction. A semi-automatic procedure for axial skeleton VOI definition is described in a previous study which shows high reproducibility 23. A software XD3 (Mirada Medical) was used for PET-CT image display and processing. The VOI including the spine and the pelvis was firstly determined by CT densities of Hounsfield units >130, and then all irrelevant bone areas were manually excluded. The final CT VOIs were then displayed on fused PET images to check if there were possible regions of increased ^18^F-FDG uptake near the skeleton, including extramedullary lesions and bladder. Areas of contiguous bone involvement and bone hyperplasia and sclerosis were also manually excluded.

From PET/CT VOIs, in total 1826 quantitative features including 781 features from PET and 1045 from CT were extracted. We extracted the radiomics features with the PyRadiomics package 24 (https://github.com/Radiomics/pyradiomics) which is compliant with the Imaging Biomarker Standardization Initiative 25. From this package, we extracted the radiomics features from the original PET and CT images, filtered images with coiflet wavelet and Laplacian of Gaussian (LoG) respectively. The images were discretized with a fixed bin size of 25 HU, which was quite commonly used in radiomics literature 26-28. The extracted features reflected the disease characteristics including intensity distribution, texture pattern, morphological information, and spatial locations, as well as wavelet features 24. The detailed list of extracted features was provided in the Supplementary Materials (I. Experimental settings of radiomic features). Conventional PET metrics were also considered with equivalent features included in the features list. Specifically, the maximum and mean of the standard uptake value (SUV) were represented by the “Intensity Histogram” features “Maximum” and “Mean” from the original PET image, and the metabolic tumor volume (MTV) could be represented by “Morphology” feature “Volume”. Texture patterns were represented statistically by some common matrix, such as gray level co-occurrence matrix (GLCM), gray level size zone matrix (GLSZM), and gray level run length matrix (GLRLM). In addition, features from LoG and wavelet images were able to depict subtle texture features at different coarseness levels and frequency domains.

The second stage was important feature selection with model construction. To reduce the high dimensionality of features, our selection strategy incorporated both intrinsic and statistical feature relationship as well as an outcome-driven machine learning model. To ensure that the feature-set was accurately clustered, we first repeated consensus cluster sampling for n=50 times to achieve the most stable groups. And then, to select the most important features, our selection process included: 1) from each cluster, the most representative features were selected based on random forest 29 tree importance (importance ≥ 0.01), 2) key features were selected from the representative features by univariate random forest using the area under the curve (AUC ≥ 0.7), 3) to further eliminate the remaining redundant features, we then utilized the pairwise Pearson correlation matrix, 4) recursive feature elimination 30 was adopted to select the most important features to form radiomic pattern. Thereby, the machine learning prediction model could be constructed only with the selected important features using Random Forest algorithm. The detailed settings of Random Forest are provided in the Supplementary Materials (II. Parameters setting of the Random Forest prediction model).

The last stage was model validation. The machine learning model was trained by a Stratified ten-fold cross-validation on the training dataset, and the proportion of the positive-negative sample ratio in training and testing sets were approximately the same as in the original data set. To validate the robustness and stability of the machine learning model, we utilized both cross-validations and independent validations to assess the performance of the model. Ten-fold cross-validations were performed within the training group. As to the independent validations, the model was trained with the entire training group and then evaluated on the independent validation group. Feature importance ranking were adopted in the random forest model to evaluate the representative value of selected features. The feature-set was continuously and randomly permuted and scored, and the importance scores of the variable were obtained.

The performance of the pattern in this model was evaluated using receiver operating characteristic (ROC) curve. Wilcoxon test was utilized for feature *P* values (*P* ≤ 0.05) selection for both continuous and classification variables. The sensitivity, specificity, accuracy, positive predictive value (PPV) and negative predictive value (NPV) were also computed by Confusion matrix-derived metrics. Statistical analyses were performed “scikit-learn”, “scipy”, “math” packages in Python programming language.

## Results

### Clinical visual analysis

The visual analysis was performed on the patients of training group with 16 BMB positive and 19 BMB negative patients. According to the visual analysis, 3 patients were classified as focal uptake, 8 as normal uptake and 24 patients were classified as diffuse uptake. Visual analysis correctly diagnosed all focal uptake patients and 7 out of 8 normal uptake patients. However, as to the diffuse uptake cases, visual analysis correctly diagnosed 14 cases, with 7 TP and 7 TN, failed in 10 cases with 5 FP and 5 FN. In summary, visual analysis achieved a successful diagnosis in 68.6% (24/35) of patients. The AUC of the visual analysis was 0.681 (95% confidence interval was 0.502-0.828). Its sensitivity, specificity, accuracy, PPV and NPV was 62.5%, 73.7%, 68.6%, 66.7% and 70.0%, respectively.

### Feature selection and machine learning model

**Figure 2:**
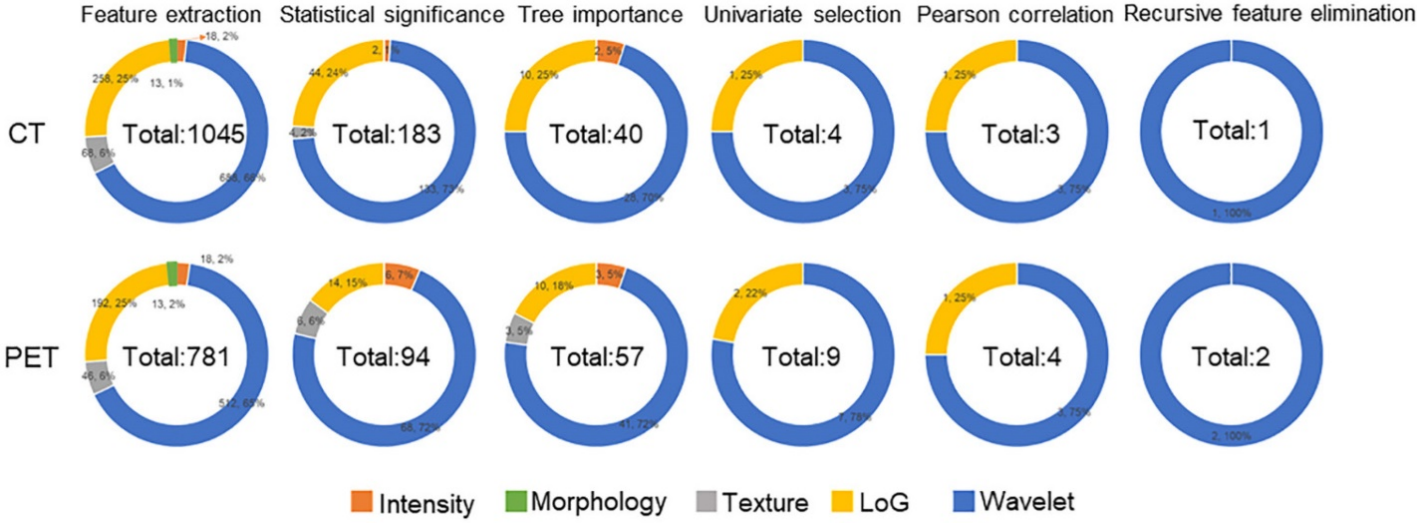
The results of feature reductions.

Feature selection procedure and results are illustrated as **Figure 2**. It could be observed that although Morphology features were extracted from images, these features were eliminated due to their statistical insignificance by statistical analysis. The texture features from original CT image were all eliminated due to their less importance determined by the Random Forest algorithm. The following feature univariate random forest selection showed that the features from original PET and CT images were less predictive in comparison with the features from LoG filtered and Wavelet decomposed images. Finally, after recursive feature elimination process, the machine learning model consisted of two PET and one CT features (**Table 2**). It could be observed that the three selected features were all from the wavelet decomposed images capturing the textural information with low pass filters applied to the first two dimensions and high pass filter applied to the last dimension. The feature values extracted from the experimental dataset are normalized and summarized in **Table 3**. These values were assigned different weights when performing the model prediction.

The model was evaluated with both cross- validation and independent validation. In the cross- validation, the model correctly predicted 31 patients with 14 TP and 17 TN, incorrectly predicted 4 (2 FP and 2 FN) patients of ^18^F-FDG diffuse uptake. The machine learning model achieved a successful diagnosis in 88.6% (31/35) of patients, which was significantly higher than that of visual analysis by using Pearson Chi-square test (*P*=0.041). The AUC of the model was 0.885 (95% confidence interval was 0.732-0.968), which was significantly higher than that of visual analysis (*P*=0.046). Its sensitivity, specificity, accuracy, PPV and NPV was 87.5%, 89.5%, 88.6%, 87.5% and 89.5%, respectively. As to the independent validations, the prediction model could achieve 83.3% (5/6) accuracy on the independent validation dataset. Among the six patients, one (out of two) focal uptake patient was incorrectly predicted as FN, while all the diffuse uptake and normal uptake patients were correctly predicted.

### Results analysis and interpretation

Results from the study show that the differences between the two methods mainly existed in the diagnosis of the patients with diffuse uptake. The machine learning model achieved 83.3% (20/24) prediction accuracy, in comparison with 58.3% (14/24) accuracy from visual analysis. Among the 10 visually failed diffuse uptake cases, the machine learning model correctly predicted 9 of them. Visual analysis correctly diagnosed the other three cases in which the machine learning model failed.

**Figure 3:**
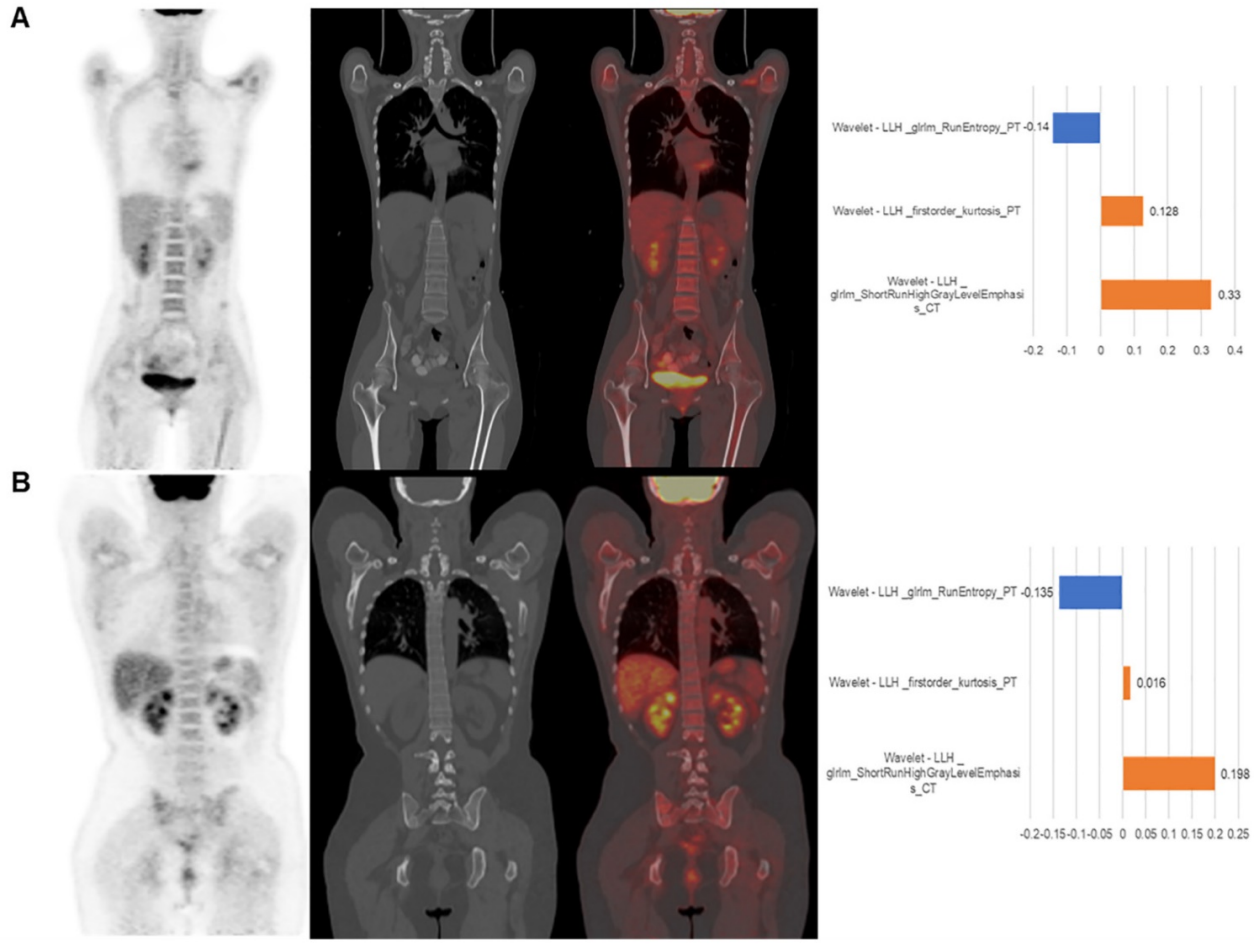
The patient displayed on panel (A) was BMB negative. The machine learning model correctly predicted it. The value of three features were -0.03, -0.57 and -0.23, respectively. The visual diagnosis was false positive. The patient displayed on panel (B) was BMB positive. The machine learning model correctly predicted it. The value of the three features were -0.48, -0.34 and -0.28, respectively. The visual diagnosis was false negative. From left to right, coronal PET, CT, fusion image and the approximated features weights from LIME interpretation.

**Figure 4:**
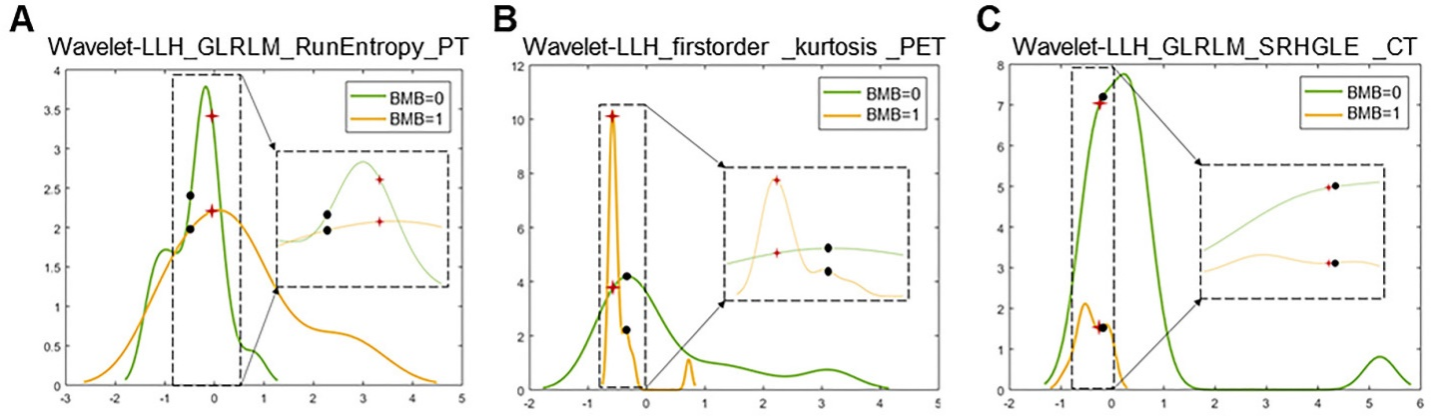
Distribution histograms (feature values as x-axis, and value frequency in the dataset as y-axis) of the 3 radiomic features selected by the trained machine learning model with the corresponding feature values of the 2 representative cases (red crosses for case 3A, and black spots for case 3B). The zoomed and scaled views of the distributions are indicated with dashed frames (BMB=0 for bone marrow biopsy negative, and BMB=1 for bone marrow biopsy positive).

Two representative cases from visual analysis and machine learning model are illustrated by **Figure 3**. As shown in **Figure 4** for the distribution histogram of the three normalized features among all experimental data, there existed BMB positive and BMB negative patients sharing same feature value ranges. Therefore, BMB positive and negative patients could not be discriminated from an individual feature (with mean accuracy of 70.8%, 72.7% and 76.7% respectively for Kurtosis, RunEntropy and SRHGLE features). As to the case **3A**, according to the first and third features, since there were more BMB negative patients than positive ones exhibiting the same feature value, these two features would suggest that the patient was more probably to be BMI negative. However, the distribution of the second feature was against this negative suggestion. As to the case **3B**, although all three features were suggesting that the patient was more likely to be negative, the possibility of a positive case could not be eliminated, given that a few positive cases were exhibiting the same feature values.

The machine learning model quantitatively combined these features for the final prediction while considering their diverse contributions. The contributions of features could be explained by the weighting coefficients derived from Local Interpretable Model- agnostic Explanations (LIME) model which is a local linear approximation of the trained prediction model 31. The LIME model perturbed the feature values and observed the resulted changes in prediction. The features, which the prediction was more sensitive to, would be assigned higher weight values. Positive weights indicated that the increase in the corresponding features would be more supporting a positive prediction, while negative weights would indicate the changes supporting a negative prediction. The right column of **Figure 3** shows the features weights employed in the prediction of the two representative cases, and the predictions were derived from the linear combinations of the features weights and features values.

## Discussion

To tackle the well-recognized difficulties of visual analysis of BMI, we developed a ^18^F-FDG PET/CT radiomic analysis in the patients with suspected relapsed AL. To the best of our knowledge, there have been no previous studies using radiomic features with machine learning methods to assess leukemic bone marrow uptake, and it is a relatively large-scale bone marrow^18^F-FDG PET/CT study.

Considering the sample size, we employed the Random Forest prediction model in our study. As evaluated by Gunduz et al 32, the random forest model substantially outperformed other techniques on both real life and simulated data regarding the task of robust classification in the high dimension low sample size context. Floares et al 33 further justified that the Random Forest method would derive accurate and robust model from omics data of small sample size. Such characteristic made random forest model more suitable to our study where radiomic pattern would be derived from high dimensional data (a total of 1826 features for each patient) of limited number of sample studies. Additionally, according to the theory of Chalkidou et al 34, 10 to 15 patients are minimally required to test one radiomic feature, our model reduced the number of features to 3 features and would be valid to minimize false detection rates regarding the sample size in our study. The intra- and inter-observer variabilities and their influence on the performance of our prediction model was also evaluated in the study in Supplementary Materials (III: Influence of intra- and inter-observer variability on prediction).

The first finding of this study is that the machine learning model achieved a high accuracy for detecting the BMI, outperforming that of visual analysis, and was particularly excellent in analyzing diffuse uptake patterns. The diagnostic value of machine learning model statistically outperformed visual analysis in terms of AUC (0.885 vs. 0.681, *P*=0.046), and the successful diagnosis rate of machine learning model was significantly higher than that of visual analysis (88.6% vs. 68.6%, *P*=0.041). For the diffuse uptake patients, the machine learning model achieved 83.3% (20/24) prediction accuracy, in comparison with 58.3% (14/24) accuracy from visual analysis. The independent validation further justified the excellence of the machine learning model for diffuse uptake pattern. To the best of our knowledge, this is the first study to apply artificial intelligence technology to improve the ^18^F-FDG PET/CT-based clinical diagnosis of BMI in the patients with suspected relapsed AL. A comparable radiomic analysis result was reported in the patients with diffuse large B cell lymphoma, where the AUC of a first-order Skewness feature in detecting BMI was 0.821, and its sensitivity and specificity was 81.8% and 81.7%, respectively 23. The Skewness feature and its variants were also extracted in our experiments, and their performances (mean accuracy of 52%, range 34.7%~67.2%) were all lower than that of the individual three features we selected, and thereby also lower than the performance of our radiomic pattern (Supplementary Materials IV. Comparison of Skewness features with selected features).

Another finding is that this study provided an interpretable insight into the output of BMI from the machine learning model. Due to the complexity and opacity of algorithms, machine learning methods are often criticized as black boxes. We attempted to interpret the results of model predictions based on the LIME model. LIME approximated the machine learning model as a local linear model which is a linear combination of the feature values and the corresponding relative weighting coefficients. With the derived weights of features, the driving factors of the machine learning model prediction could be extracted. A more detailed explanation is in the results section.

Interestingly, a CT feature became an integral part of the model in the present study. Although the value of features extracted from unenhanced low- dose CT has been demonstrated in the studies of non-small cell lung cancer 35, lymphoma 36 and esophageal cancer 37, there are no such published studies on bone marrow. Based on the experience of visual analysis, CT is suitable to visualize cortical and trabecular bone, while not a routine method for bone marrow assessment 38,39. In the present study, the CT feature contributed with a relatively high weight in some patients. However, the value of CT features on BMI requires a larger number of research samples for further confirmation.

In addition, in comparison to the PET conventional metrics (SUVmax, SUVmean, MTV and TLG), our selected radiomics features possessed much stronger correlations with BMB. The equivalent features to the three conventional metrics, i.e. SUVmax, SUVmean and MTV, were initially included in the extracted radiomics set. However, these three equivalent features were excluded automatically by our feature selection procedure on the basis of their discriminative contributions. We calculated another conventional metric, TLG=MTV*SUVmean 40. The prediction accuracy for these four individual conventional metrics were 53.9%, 44.2%, 50.5% and 51.5% respectively. Further comparison analysis on the correlations with BMB was performed between PET conventional metrics and our three selected radiomics features (Table 4). The comparison showed that the BMB correlation values of our selected radiomics features were 0.42, -0.41 and -0.38 while the correlation values of the four PET conventional metrics were -2.33E-01, 0.19, 0.22 and 0.29.

The last finding is that our automated radiomic analysis method could serve as a non-invasive test option complementing the visual analysis for the diagnosis of suspected relapsed AL. For the 11 failed cases in visual analysis, our machine learning model correctly predicted 10 of them by analyzing the radiomic features purely based on the PET/CT scans. And that would suggest our model being an eligible non-invasive test option complementing the visual analysis for a more comprehensive and accurate diagnosis.

For the next stage, we will be performing translational research by 1) harnessing automated bone segmentation software with machine learning based prediction model for automated processing and analysis platform, and 2) installing the software platform in our collaborative hospitals for multi- center study for standardization of the imaging biomarkers for BMB.

## Conclusion

^18^F-FDG PET/CT radiomic analysis with machine learning model provided an objective and efficient mechanism for identifying the BMI in suspected relapsed AL, and could serve as a non-invasive test option complementing the visual analysis to derive a more comprehensive, confident and accurate diagnosis. It is suggested in particular for the diagnosis of BMI in the patients with diffuse uptake.

## Supplementary Material

Supplementary information, figures and tables.

## Figures and Tables

**Table 1:** Demographic and clinical characteristics of patients.

Characteristics	Total population	BMB positive	BMB negative	*P* value
	(*n*=41)	(*n* =18)	(*n* =23)	
Age (years), median (range)	35.2 (17~75)	38.1 (18~75)	32.9 (17~49)	0.276
Gender (female/ male)	15/ 26	4/ 14	11/ 12	0.089
Leukemia subtype (ALL/ AML)	17/ 24	5/ 13	12/ 11	0.116
With extramedullary relapse/ without	24/ 17	11/ 7	13/ 10	0.767
**Laboratory parameters**				
WBC (G/L), mean (SD)	6.62 (4.70)	8.19 (6.27)	5.35 (2.38)	0.092
Hb (g/dL), mean (SD)	114.45 (23.07)	111.10 (21.53)	117.17(24.42)	0.427
ESR (mm/h), mean (SD)	38.33 (26.45)	37.17 (22.16)	39.50 (32.32)	0.887
CRP (mg/L), mean (SD)	13.01 (21.92)	18.32 (28.59)	7.08 (7.21)	0.199

ALL: acute lymphoblastic leukemia, AML: acute myeloid leukemia, WBC: white blood cell, Hb: hemoglobin, ESR: erythrocyte sedimentation rate, CRP: C reaction protein

**Table 2:** The features selected from the trained machine learning model and their meanings.

Feature name	Feature definition and meaning
Wavelet-LLH_GLRLM_RunEntropy_PET	Formula: 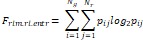 Where 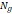 is the number of discretized grey level intensity in the mask of VOI, 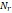 is the maximal possible run length in the mage. 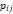 is normalized the run length matrix. 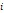 is a discretized grey level and 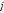 is occurrences of runs with length in matrix. Measuring the distribution of gray levels randomness from an image filter by a mid-frequency wavelet. The higher the value, the stronger the heterogeneity in the texture patterns.
Wavelet-LLH_firstorder _kurtosis _PET	Formula: 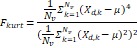 Where 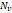 is the intensities set included in the ROI intensity mask denoted as { 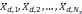 }. 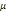 is average gray level intensity within the VOI, Measuring the peak of image VOI pixel value distribution in a mid-decomposition domain by using wavelet filter. The lower the value implies the mass of distribution concentrated towards a peak close to the mean value, vice versa.
Wavelet-LLH_GLRLM_SRHGLE _CT	Formula: 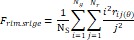 Where 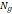 is the number of discretized grey level intensity in the mask of VOI, 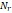 is the maximal possible run length in the mage. 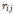 be the run length matrix for an arbitrary direction 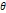 . 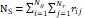 be the number of runs in the image along angle 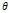 . 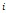 is a discretized grey level and 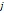 is occurrences of runs with length in matrix. Measuring the distribution of homogeneity by measuring the short run length distribution of higher gray values after mid-pass wavelet filter.

GLRLM: gray level run length matrix, LLH: low, low, and high frequency, SRHGLE: short run high gray level emphasis

**Table 3:** The mean± standard (SD), range and *P* value of the features of the BMB positive and negative patients.

	BMB positive			BMB negative		*P* value
	Mean± SD	Range		Mean± SD	Range	
Wavelet-LLH _GLRLM_RunEntropy_PET	0.453±1.190	-1.02~3.09		-0.381±0.528	-1.26 ~0.81	0.022
Wavelet - LLH _firstorder_kurtosis _PET	-0.443±0.320	-0.62~0.72		0.375±1.183	-0.61~3.21	0.008
Wavelet-LLH_ GLRLM_SRHGLE_ CT	-0.404±0.273	-0.93~-0.04		0.34 ±1.24	-0.69~5.21	0.001

GLRLM: gray level run length matrix, LLH: low, low, and high frequency, SRHGLE: short run high gray level emphasis

**Table 4:** The correlation matrix of selected features, PET conventional metrics and BMB.

Features	SUVmax	SUVmean	MTV	TLG	BMB
Wavelet-LLH_GLRLM_RunEntropy_PET	-6.63E-02	0.85	0.24	0.89	**0.42**
Wavelet-LLH_firstorder_Kurtosis_PET	6.00E-01	-0.24	-0.16	-0.22	**-0.41**
Wavelet-LLH_GLRLM_SRHGLE_CT	-2.70E-03	0.04	-0.45	0.1	**-0.38**
BMB	**-2.33E-01**	**0.19**	**0.22**	**0.29**	1

## Abbreviations

AL: acute leukemia
ALL: acute lymphoblastic leukemia
AML: acute myeloid leukemia
AUC: area under the curve
BMB: bone marrow biopsy
BMI: bone marrow involvement
CRP: C reaction protein
ESR: erythrocyte sedimentation rate
^18^F-FDG PET/ CT: ^18^F-fluorodesoxyglucose positron emission tomography/computed tomography
FN: false negative
FP: false positive
GLCM: gray level co-occurrence matrix
GLRLM: gray level run length matrix
GLSZM: gray level size zone matrix
LoG: Laplacian of Gaussian
LIME: local interpretable model-agnostic explanations
MTV: metabolic tumor volume
NPV: negative predictive value
PPV: positive predictive value
RBC: red blood cell
ROC: receiver operating characteristic
SUV: standard uptake value
TN: true negative
TP: true positive
VOI: volume of interest
WBC: white blood cell

## References

[1] de Lima M, Porter DL, Battiwalla M, Bishop MR, Giralt SA, Hardy NM (2014). Proceedings from the National Cancer Institute's Second International Workshop on the Biology, Prevention, and Treatment of Relapse After Hematopoietic Stem Cell Transplantation: part III. Prevention and treatment of relapse after allogeneic transplantation. Biology of Blood and Marrow Transplantation.

[2] Percival ME, Lai C, Estey E, Hourigan CS (2017). Bone marrow evaluation for diagnosis and monitoring of acute myeloid leukemia. Blood Rev.

[3] Cistaro A, Saglio F, Asaftei S, Fania P, Berger M, Fagioli F (2011). The role of 18F-FDG PET/CT in pediatric lymph-node acute lymphoblastic leukemia involvement. Radiol Case Rep.

[4] Stolzel F, Rollig C, Radke J, Mohr B, Platzbecker U, Bornhauser M (2011). 18F-FDG-PET/CT for detection of extramedullary acute myeloid leukemia. Haematologica.

[5] Cribe AS, Steenhof M, Marcher CW, Petersen H, Frederiksen H, Friis LS (2013). Extramedullary disease in patients with acute myeloid leukemia assessed by 18F-FDG PET. Eur J Haematol.

[6] Zhou WL, Wu HB, Wang LJ, Tian Y, Dong Y, Wang QS (2016). Usefulness and pitfalls of F-18-FDG PET/CT for diagnosing extramedullary acute leukemia. Eur J Radiol.

[7] Elojeimy S, Luana Stanescu A, Parisi MT (2016). Use of 18F-FDG PET-CT for Detection of Active Disease in Acute Myeloid Leukemia. Clinical nuclear medicine.

[8] Cunningham I, Kohno B (2016). 18 FDG-PET/CT: 21st century approach to leukemic tumors in 124 cases. Am J Hematol.

[9] Su K, Nakamoto Y, Nakatani K, Kurihara K, Hayakawa N, Togashi K (2013). Diffuse homogeneous bone marrow uptake of FDG in patients with acute lymphoblastic leukemia. Clinical nuclear medicine.

[10] Parida GK, Soundararajan R, Passah A, Bal C, Kumar R (2015). Metabolic Skeletal Superscan on 18F-FDG PET/CT in a Case of Acute Lymphoblastic Leukemia. Clinical nuclear medicine.

[11] Arimoto MK, Nakamoto Y, Nakatani K, Ishimori T, Yamashita K, Takaori-Kondo A (2015). Increased bone marrow uptake of 18F-FDG in leukemia patients: preliminary findings. Springerplus.

[12] Su Z, Wu F, Hu W, Liu X, Wu S, Feng X (2017). Philadelphia chromosome-positive acute myeloid leukemia with masses and osteolytic lesions: finding of 18F-FDG PET/CT. Frontiers of medicine.

[13] Adams HJ, Nievelstein RA, Kwee TC (2015). Opportunities and limitations of bone marrow biopsy and bone marrow FDG-PET in lymphoma. Blood Rev.

[14] Salaun PY, Gastinne T, Bodet-Milin C, Campion L, Cambefort P, Moreau A (2009). Analysis of 18F-FDG PET diffuse bone marrow uptake and splenic uptake in staging of Hodgkin's lymphoma: a reflection of disease infiltration or just inflammation?. Eur J Nucl Med Mol Imaging.

[15] Knopp MV, Bischoff H, Rimac A, Oberdorfer F, van Kaick G (1996). Bone marrow uptake of fluorine-18-fluorodeoxyglucose following treatment with hematopoietic growth factors: initial evaluation. Nuclear medicine and biology.

[16] Berthet L, Cochet A, Kanoun S, Berriolo-Riedinger A, Humbert O, Toubeau M (2013). In newly diagnosed diffuse large B-cell lymphoma, determination of bone marrow involvement with 18F-FDG PET/CT provides better diagnostic performance and prognostic stratification than does biopsy. J Nucl Med.

[17] Adams HJ, Kwee TC, Fijnheer R, Dubois SV, Nievelstein RA, de Klerk JM (2015). Bone marrow FDG-PET/CT in Hodgkin lymphoma revisited: do imaging and pathology match?. Ann Nucl Med.

[18] Adams HJ, Kwee TC, Fijnheer R, Dubois SV, Nievelstein RA, de Klerk JM (2014). Bone marrow 18F-fluoro-2-deoxy-D-glucose positron emission tomography/computed tomography cannot replace bone marrow biopsy in diffuse large B-cell lymphoma. Am J Hematol.

[19] Soydal C, Koksoy EB, Yasar A, Turgal E, Erdogan BD, Akbulut H (2016). Prognostic Importance of Bone Marrow Uptake on Baseline 18F-FDG Positron Emission Tomography in Diffuse Large B Cell Lymphoma. Cancer Biother Radiopharm.

[20] Antunovic L, Gallivanone F, Sollini M, Sagona A, Invento A, Manfrinato G (2017). [18F]FDG PET/CT features for the molecular characterization of primary breast tumors. Eur J Nucl Med Mol Imaging.

[21] Lucia F, Visvikis D, Desseroit MC, Miranda O, Malhaire JP, Robin P (2018). Prediction of outcome using pretreatment 18F-FDG PET/CT and MRI radiomics in locally advanced cervical cancer treated with chemoradiotherapy. Eur J Nucl Med Mol Imaging.

[22] Sollini M, Cozzi L, Antunovic L, Chiti A, Kirienko M (2017). PET Radiomics in NSCLC: state of the art and a proposal for harmonization of methodology. Sci Rep.

[23] Aide N, Talbot M, Fruchart C, Damaj G, Lasnon C (2018). Diagnostic and prognostic value of baseline FDG PET/CT skeletal textural features in diffuse large B cell lymphoma. Eur J Nucl Med Mol Imaging.

[24] van Griethuysen JJM, Fedorov A, Parmar C, Hosny A, Aucoin N, Narayan V (2017). Computational radiomics system to decode the radiographic phenotype. Cancer research.

[25] Zwanenburg A, Leger S, Vallières M, Löck S Image biomarker standardisation initiative. 2018. arXiv preprint ar Xibv:1612.07003.

[26] Welch ML, McIntosh C, Haibe-Kains B, Milosevic MF, Wee L, Dekker A (2019). Vulnerabilities of radiomic signature development: The need for safeguards. Radiotherapy and Oncology.

[27] Dou TH, Coroller TP, van Griethuysen JJ, Mak RH, Aerts HJ (2018). Peritumoral radiomics features predict distant metastasis in locally advanced NSCLC. PloS one.

[28] Yuan R, Shi S, Chen J, Cheng G (2018). Radiomics in RayPlus: a Web-Based Tool for Texture Analysis in Medical Images. Journal of Digital Imaging.

[29] Breiman L (2001). Random Forests. Machine Learning.

[30] Guyon I, Weston J, Barnhill S, & Vapnik V (2002). Gene selection for cancer classification using support vector machines.

[31] Ribeiro MT, Singh S, Guestrin C (2016). "Why should I trust you?": Explaining the predictions of any classifier. Proceedings of the 22nd ACM SIGKDD International Conference on Knowledge Discovery and Data Mining.

[32] Gunduz N, Fokoue E (2015). Robust classification of high dimension low sample size data.

[33] Floares A, Ferisgan M, Onita D, Ciuparu A, Calin G, Manolache F (2017). The smallest sample size for the desired diagnosis accuracy.

[34] Chalkidou A, O'Doherty MJ, Marsden PK (2015). False discovery rates in PET and CT studies with texture features: A Systematic Review. PLoS ONE.

[35] Win T, Miles KA, Janes SM, Ganeshan B, Shastry M, Endozo R (2013). Tumor heterogeneity and permeability as measured on the CT component of PET/CT predict survival in patients with non-small cell lung cancer. Clinical cancer research: an official journal of the American Association for Cancer Research.

[36] Ganeshan B, Miles KA, Babikir S, Shortman R, Afaq A, Ardeshna KM (2017). CT-based texture analysis potentially provides prognostic information complementary to interim fdg-pet for patients with hodgkin's and aggressive non-hodgkin's lymphomas. Eur Radiol.

[37] Ganeshan B, Skogen K, Pressney I, Coutroubis D, Miles K (2012). Tumour heterogeneity in oesophageal cancer assessed by CT texture analysis: preliminary evidence of an association with tumour metabolism, stage, and survival. Clin Radiol.

[38] Vinnicombe SJ, Reznek RH (2003). Computerised tomography in the staging of Hodgkin's disease and non-Hodgkin's lymphoma. European Journal of Nuclear Medicine and Molecular Imaging.

[39] Kwee TC, de Klerk JM, Nievelstein RA (2011). Imaging of bone marrow involvement in lymphoma: state of the art and future directions. ScientificWorldJournal.

[40] Choi ES, Ha SG, Kim HS, Ha JH, Paeng JC, Han I (2013). Total lesion glycolysis by 18 F-FDG PET/CT is a reliable predictor of prognosis in soft-tissue sarcoma. Eur J Nucl Med Mol Imaging.

