# Supplementary material for: 18F-FDG PET/CT Radiomic Analysis with Machine Learning for Identifying Bone Marrow Involvement in the Patients with Suspected Relapsed Acute Leukemia
Source: Theranostics. 2019 Jul 9;9(16):4730–9. doi: 10.7150/thno.33841 (PMC6643435; doi:10.7150/thno.33841)
Supplement: Supplementary file 1 — Supplementary information, figures and tables. [file thnov09p4730s1.pdf]

# Supplementary Materials


I. **Experimental settings of radiomic features extraction**

II. **Parameters setting of the Random Forest prediction model**

III. **Comparison of Skewness features with our selected features**

IV. **Influence of intra- and inter-observer variability on prediction**

V. **References**

## Experimental settings of radiomic features extraction

**Table 1** lists the radiomics features we extracted from the original PET and CT images, while the 22 GLCM features were only extracted from CT images. The definitions of these features were compliant with the Image Biomarker Standardisation Initiative [1].

In addition to features from original PET and CT images, we also extracted the same set of radiomics features respectively from the LoG and wavelet filtered images of both PET and CT images. The LoG filters were applied with three levels, i.e. sigma=1mm, 3mm and 5mm, capturing three different levels of texture details. The wavelet filters could yield 8 decomposed images by applying all possible combinations of high or low pass filter in each of the three dimensions. The **Figure 1** shows the distribution of all radiomics features after feature extraction, and the detailed parameters settings for these radiomics feature extraction could be found in **Table 2**.

**Table 1**. The full list of extracted radiomic features.

| Intensity histogram(18) | Morphology(13) | GLCM(22) | GLRLM(16) | GLSZM(16) | NGTDM(14) |
|---|---|---|---|---|---|
| 10percentile | Elongation | AutoCorrelation | GLNU | GLNU | DependenceEntropy |
| 90Percentile | Flateness | ClusterProminence | GLNUN | GLNUN | DNU |
| Energy | LeastAxis | ClusterShade | HGLRE | GLV | DNUN |
| Entropy | MajorAxis | ClusterTendency | LRE | HGLZE | DependenceVariance |
| InterquartileRange | M2DDC | Contrast | LRHGLE | LAE | GLNU |
| Kurtosis | M2DDR | Correlation | LRLGLE | LAHGE | GLV |
| Maximum | M2DDS | DifferenceAverage | LGLRE | LALGE | HGLE |
| Mean | M3DD | DifferenceEntropy | RunEntropy | LGLZE | LDE |
| MAD | MinorAxis | DifferenceVariance | RLNU | SZNU | LDLGLE |
| Median | Sphericity | Id | RLNUN | SZNUN | LDHGLE |
| Range | SurfaceArea | Idm | RunPercentage | SAE | LGLE |
| RMAD | SVR | Idmn | RunVariance | SAHGE | SDE |
| RMS | Volume | Idn | SRE | SALGE | SDHGE |
| TotalEnergy | | Imc1 | SRHGE | ZoneEntropy | SDLGE |
| Uniformity | | Imc2 | SRLGE | ZonePercentage | |
| Variance | | InverseVariance | GLV | ZoneVariance | |
| Minimum | | JointAverage | | | |
| Skewness | | JointEnergy | | | |
| | | JointEntropy | | | |
| | | MaxProbability | | | |
| | | SumEntropy | | | |
| | | SumSquares | | | |

GLCM: gray level cooccurrence matrices; GLRLM: gray level run length matrix; GLSZM: gray level size zone matrix; NGTDM: neighborhood gray-tone difference matrix wavelet decompositions.

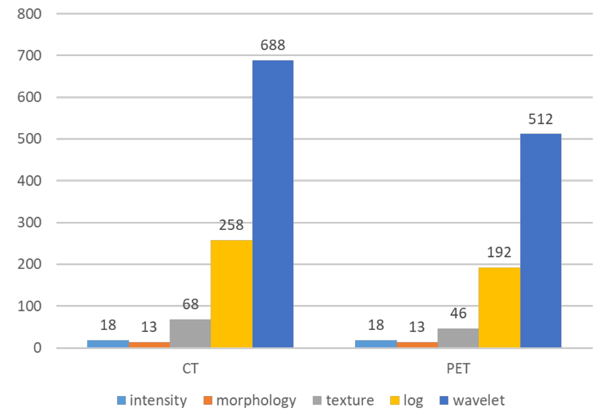


**Figure 1**. The distribution of extracted features.

**Table 2**. The parameter settings for features extraction.

<table>
<tr><th>Image type</th><th>Original</th><th>LoG</th><th>Wavelet(“coif”)</th></tr>
<tr><td>Intensity</td><td>voxelArrayShift: 1000<br>padDistance = 10</td><td rowspan="5">Sigma=1mm<br>Sigma=3mm<br>Sigma=5mm</td><td rowspan="5">LLL, LLH, LHL,<br>LHH, HHH, HLL,<br>HLH, HHL</td></tr>
<tr><td>GLCM</td><td>Distance=1<br>symmetricalGLCM = True<br>weightingNorm =None</td></tr>
<tr><td>GLRLM</td><td>weightingNorm =None</td></tr>
<tr><td>GLDM(NGLDM)</td><td>Distance=1<br>Gldm_a=0</td></tr>
<tr><td>GLSZM</td><td></td></tr>
<tr><td>Morphology</td><td></td><td></td><td></td></tr>
</table>

### Parameters setting of the Random Forest prediction model

The final prediction model was trained by the Random Forest algorithm with only the three selected important features, and the parameters of the model are listed in **Table 3**.

**Table 3**. The parameters of RandomForest model.

| | |
|---|---|
| bootstrap | TRUE |
| class_weight | None |
| criterion | 'gini' |
| max_depth | None |
| max_features | 'auto' |
| max_leaf_nodes | None |
| min_impurity_decrease | 0 |
| min_impurity_split | None |
| min_samples_leaf | 1 |
| min_samples_split | 2 |
| min_weight_fraction_leaf | 0 |
| n_estimators | 100 |
| n_jobs | 1 |
| oob_score | FALSE |
| random_state | 24 |
| verbose | 0 |
| warm_start | FALSE |

### Comparison of Skewness features with our selected features

The radiomic study of diffuse large B cell lymphoma [2] reported the diagnostic and prognostic value of the first-order Skewness feature in detecting BMI. However, the Skewness feature was not credible regrading the identification of the BMI in suspected relapsed AL. As recorded in **Table 4**, the Skewness feature along with its variants in cross-validation achieved mean accuracy of 52% (range 34.7%~67.2%) which was much lower than the individual performance of the three features we selected.

**Table 4**. The individual feature prediction performance of RunEntropy, Kurtosis, SRHGLE and Skewness.

| | Features | Mean accuracy |
|---|---|---|
| Our selections | Kurtosis_wavelet_LLH.PET | 0.708 |
| | RunEntropy_wavlet_LLH.PET | 0.727 |
| | SRHGLE_wavelet_LLH.CT | 0.767 |
| PET | original_firstorder_Kurtosis.PET | 0.437 |
| | original_glrlm_RunEntropy.PET | 0.577 |
| | original_glrlm_SRHGLE.PET | 0.537 |
| | Skewness_wavelet_LLH.PET | 0.487 |
| | Skewness_wavelet_LLL.PET | 0.580 |
| | Skewness_wavelet_LHL.PET | 0.530 |
| | Skewness_wavelet_LHH.PET | 0.479 |
| | Skewness_wavelet_HLL.PET | 0.489 |
| | Skewness_wavelet_HLH.PET | 0.536 |
| | Skewness_wavelet_HHL.PET | 0.563 |
| | Skewness_wavelet_HHH.PET | 0.473 |
| | Skewness_log_sigma1.PET | 0.502 |
| | Skewness_log_sigma3.PET | 0.385 |
| | Skewness_log_sigma5.PET | 0.586 |
| | Skewness_original.PET | 0.347 |
| CT | Skewness_wavelet_LLH.CT | 0.671 |
| | Skewness_wavelet_LLL.CT | 0.373 |
| | Skewness_wavelet_LHL.CT | 0.626 |
| | Skewness_wavelet_LHH.CT | 0.645 |
| | Skewness_wavelet_HLL.CT | 0.496 |
| | Skewness_wavelet_HLH.CT | 0.420 |
| | Skewness_wavelet_HHL.CT | 0.482 |
| | Skewness_wavelet_HHH.CT | 0.594 |
| | Skewness_log_sigma1.CT | 0.524 |
| | Skewness_log_sigma3.CT | 0.466 |
| | Skewness_log_sigma5.CT | 0.544 |
| | Skewness_original.CT | 0.672 |
| | original_firstorder_Kurtosis.CT | 0.411 |
| | original_glrlm_SRHGLE.CT | 0.433 |
| | original_glrlm_RunEntropy.CT | 0.537 |

**Influence of intra- and inter-observer variability on prediction**

We evaluated the influence of both intra-observer and inter-observer variabilities on the performance of our prediction model. The volume overlap error rate (VOE) was used to measure both intra-observer and inter-observer agreement rate where 1 indicating two identical VOIs and 0 indicating no overlap between the VOIs.

**Intra-observer variability**

To evaluate the influence of intra-observer variability, 16 patients were randomly picked for a second review and adjustment. As shown in **Figure 2**, the mean intra-observer agreement rate with standard deviation was $0.949 \pm 0.044$, which led to feature value differences as recorded in **Table 5**. We then performed ten-fold cross-validations on the adjusted patients VOIs. The validation demonstrated that such a minor variability did not pose alteration on prediction decision, justified by 87.5% (14/16) accuracy with two (out of 10) diffuse uptake patient predicted as FPs, which were the identical decision as that in the previous cross-validations.

**Inter-observer variability**

To evaluate the influence of inter-observer variabilities, we further collected six new patient datasets for independent validation. The six datasets included two diffuse uptake patients, two focal uptake patients and two normal uptake patients. For each new patient, two sets of VOIs were delineated by (1) the same experienced senior operator for the initial 35 patients, and (2) a team of two junior operators. As shown in **Figure 2**, the inter-observer agreement rate was $0.921 \pm 0.074$, leading to feature value differences as recorded in **Table 5**. Then, we respectively performed independent validations on the two set of VOIs from the six new patients. The comparison of independent validations results demonstrated that there was no alteration on prediction decision, justified by the same accuracy of 83.3% (5/6) with one (out of two) focal uptake patients incorrectly predicted as FN, while all the diffuse uptake and normal uptake patients were correctly predicted.

In particular, for the two intra- and inter-observer cases with 82.74% and 79.23% as the lowest VOI agreement rates, the model produced consistent predictions.

**Further investigation with intentional high variations**

To further investigate the influence of higher variations, we randomly picked 13 patients from the initial 35 patients and intentionally exclude various portions of the VOI of the spinal cord to create a new set of VOIs. The agreement rate between these two sets of VOIs was $0.667 \pm 0.064$ (range $0.509 \sim 0.754$). The experimental validation showed that the majority of predictions (11 out of 13 patients) remained consistent while two predictions were inversed.

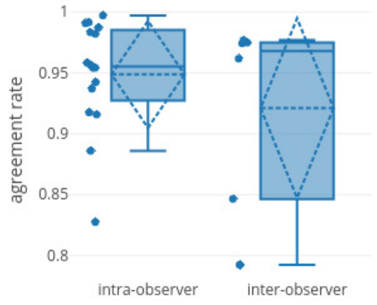


**Figure 2**. Intra-observer and inter-observer agreement rate based on VOE.

**Table 5**. Differences on normalized feature values caused by intra-observer and inter-observer variabilities.

| | Intra-observer Cases | | Inter-observer Cases | |
|---|---|---|---|---|
| | Mean± SD | Range | Mean± SD | Range |
| Wavelet-LLH_GLRLM_RunEntropy_PET | 0.018±0.036 | -0.066~0.092 | -0.001±0.069 | -0.11~0.1 |
| Wavelet-LLH_firstorder_kurtosis_PET | -0.012±0.475 | -0.787~1.624 | 0.03±0.136 | -0.1~0.28 |
| Wavelet-LLH_GLRLM_SRHGLE_CT | -0.003±0.012 | -0.007~0.048 | -0.006±0.008 | -0.02~0 |

**References**

1. Zwanenburg A, Leger S, Vallières M, Löck S. Image biomarker standardisation initiative. 2018. arXiv preprint ar Xibv:1612.07003.

2. Aide N, Talbot M, Fruchart C, Damaj G, Lasnon C. Diagnostic and prognostic value of baseline FDG PET/CT skeletal textural features in diffuse large B cell lymphoma. Eur J Nucl Med Mol Imaging. 2018;45:699-711.
